# Full-Duplex Cooperative Sensing for Spectrum-Heterogeneous Cognitive Radio Networks

**DOI:** 10.3390/s17081773

**Published:** 2017-08-02

**Authors:** Peng Liu, Wangdong Qi, En Yuan, Li Wei, Yuexin Zhao

**Affiliations:** Department of Network Engineering, P. L. A. Army Engineering University, Nangjing 210007, China; herolp@gmail.com (P.L.); yuanen82@gmail.com (E.Y.); wlnb@hotmail.com (L.W.); zhaoyxsd@gmail.com (Y.Z.)

**Keywords:** cognitive radio networks, full-duplex radio, cooperative spectrum sensing, spectrum heterogeneity, decision fusion

## Abstract

In cognitive radio networks (CRNs), spectrum sensing is critical for guaranteeing that the opportunistic spectrum access by secondary users (SUs) will not interrupt legitimate primary users (PUs). The application of full-duplex radio to spectrum sensing enables SU to carry out sensing and transmission simultaneously, improving both spectrum awareness and CRN throughput. However, the issue of spectrum sensing with full-duplex radios deployed in heterogeneous environments, where SUs may observe different spectrum activities, has not been addressed. In this paper, we give a first look into this problem and develop a light-weight cooperative sensing framework called PaCoSIF, which involves only a pairwise SU transmitter (SU-Tx) and its receiver (SU-Rx) in cooperation. A dedicated control channel is not required for pairwise cooperative sensing with instantaneous feedback (PaCoSIF) because sensing results are collected and fused via the reverse channel provided by full-duplex radios. We present a detailed protocol description to illustrate how PaCoSIF works. However, it is a challenge to optimize the sensing performance of PaCoSIF since the two sensors suffer from spectrum heterogeneity and different kinds of interference. Our goal is to minimize the false alarm rate of PaCoSIF given the bound on the missed detection rate by adaptively adjusting the detection threshold of each sensor. We derive an expression for the optimal threshold using the Lagrange method and propose a fast binary-searching algorithm to solve it numerically. Simulations show that, with perfect signal-to-interference-and-noise-ratio (SINR) information, PaCoSIF could decrease the false alarm rate and boost CRN throughput significantly against conventional cooperative sensing when SUs are deployed in spectrum-heterogeneous environments. Finally, the impact of SINR error upon the performance of PaCoSIF is evaluated via extensive simulations.

## 1. Introduction

In cognitive radio networks (CRNs), secondary users (SUs) can access the licensed spectrum opportunistically when primary users (PUs) are not active [1]. To guarantee no interruption to legitimate PU, SUs are required to sense the presence of PU periodically. For a half-duplex cognitive radio (CR), spectrum sensing has to be scheduled sequentially with data transmission. Increasing sensing time reduces CRN capacity while increasing transmission time impairs sensing accuracy. So far, many efforts have been taken to optimize the scheduling problem [2,3,4,5,6]. Recently, the drastic progress on full-duplex radio enables SU to sense the spectrum and transmit packets simultaneously [7,8]. Full-duplex spectrum sensing can improve both spectrum awareness and CRN throughput when residual self-interference (RSI) led by full-duplex operation is sufficiently eliminated [9,10].

Current research on full-duplex sensing mainly focuses upon the impact of RSI on sensing performance as well as spectrum efficiency in the case of a single sensor [9,10,11,12,13,14,15,16] or multiple cooperative sensors [17]. It has been widely assumed that SUs are deployed in spectrum-homogeneous environments. However, recent on-site surveys reveal that spectrum heterogeneity is not unusual in practical scenarios [18]. In heterogeneous environments, SUs may have different views on spectrum activity, expressed as different signal-to-interference-and-noise-ratio (SINR) regimes [19,20]. Therefore, it is no longer safe for an SU transmitter (SU-Tx) to access the spectrum according to its own observation. Instead, an SU-Tx should cooperate with its receiver (SU-Rx) to make a link-level decision via data fusion [21].

**Figure 1:**
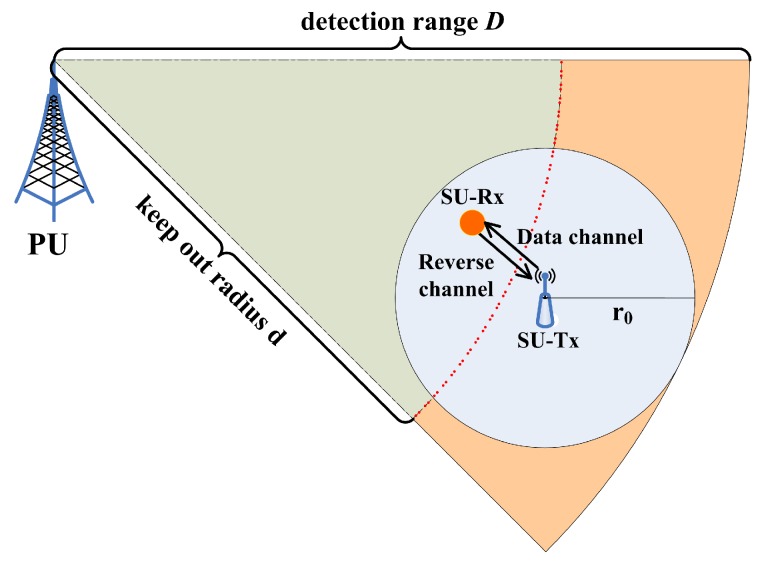
A scenario for CRN deployment in spectrum-heterogeneous environments.

In conventional cooperative spectrum sensing mechanisms, there exists a fusion center that collects the sensing data of cooperative sensors through a dedicated reporting channel [22]. Extra delay and communication overhead is incurred by the reporting process. In this paper, we propose a light-weight cooperative sensing framework for full-duplex CRN, called pairwise cooperative sensing with instantaneous feedback (PaCoSIF), to decrease data fusion overhead. In PaCoSIF, cooperative sensing is implemented only in a pairwise SU device, i.e., an SU-Tx and its SU-Rx, to meet the mandatory requirements imposed by safe spectrum access in heterogeneous environments. The SU-Tx acts as the fusion center. Before initializing a new data transmission, it starts a handshake process with the SU-Rx in the underlay mode. If the feedback from the SU-Rx confirms the spectrum availability, the SU-Tx begins data transmission in the overlay mode. Meanwhile, it collects the binary sensing results (Typically, 0 indicates PU is idle, whereas 1 means PU is active) of the SU-Rx periodically through the instantaneous reverse channel provided by full-duplex radio and then fuses them with local decisions (This is called hard decision fusion in the literature [22].), as shown in Figure 1. Since PaCoSIF does not require a dedicated control channel, the overhead is much lower against conventional cooperative sensing. A detailed protocol description to illustrate how PaCoSIF works is provided in Section 3.

The performance of an individual sensor is usually evaluated by two metrics, i.e., the missed detection rate and the false alarm rate. A missed detection event occurs when the sensor fails to detect the presence of PU, and a false alarm event occurs when the sensor reports the presence of PU incorrectly. Both of the two metrics are determined by the detection threshold. Achieving a lower missed detection rate (i.e., a higher sensitivity for PU protection) comes at the cost of a higher false alarm rate and a deteriorated CRN throughput. In the scenario of cooperative sensing, the decisions from multiple sensors are fused to relax the sensitivity requirement of an individual sensor; thus, the false alarm rate is decreased. As for hard decision fusion adopted by PaCoSIF, the most common fusion rule is the OR rule, under which PU is labeled active when any of the cooperative sensors detect the presence of PU under the OR rule. The missed detection rate of an individual sensor is relaxed equally with the OR rule [23,24]. However, this will lead to sub-optimal solutions when SUs are deployed in spectrum-heterogeneous environments and experience very different channel conditions [25].

In this paper, we try to optimize the performance of PaCoSIF under the Neyman–Pearson (NP) criterion. Our goal is to minimize the false alarm rate given the bound on the missed detection rate by adaptively adjusting the detection thresholds of the two cooperative sensors. The optimal thresholds are firstly derived using the Lagrange method when the SINR information is known a priori. Then, we propose a fast binary-searching algorithm to find numerical solutions. It is found that, when the SINR information is perfect, the solution of the binary-searching algorithm is very close to optimal. Given the same bound on the missed detection rate, the false alarm rate of PaCoSIF is much lower than that of conventional full-duplex cooperative sensing (FDCS) [17] as well as full-duplex non-cooperative sensing (FDNCS) [9] in spectrum-heterogeneous environments. Consequently, the throughput of the CRN is boosted significantly. While in homogeneous environments, the performance of PaCoSIF becomes comparable with that of FDCS and still outperforms FDNCS. Monte Carlo simulations further reveal that the cooperative gain of PaCoSIF is sensitive to the accuracy of SINR information. To be specific, the average false alarm rate of PaCoSIF will become higher as the SINR error increases. In the worst case, the SINR error misleads the optimization algorithm, and the false alarm rate of PaCoSIF may become even higher than those of FDCS or FDNCS.

The contributions of this paper can be summarized as follows:

- We design a light-weight cooperative sensing framework for full-duplex CRN deployed in sptectrum-heterogeneous environments called PaCoSIF, in which a mandatory cooperation between an SU-Tx and its SU-Rx is implemented with little overhead.
- We derive optimal detection thresholds for the sensors involved in PaCoSIF and propose a fast binary-searching algorithm to obtain numerical solutions. With perfect SINR information, the false alarm rate of PaCoSIF can be minimized.
- We investigate the performance of PaCoSIF via extensive simulations. The results reveal the quantitative impact of spectrum heterogeneity and SINR error upon the performance of PaCoSIF. The necessity of threshold optimization for cooperative sensing in spectrum-heterogeneous environments is also uncovered.

The remainder of the paper is organized as follows. In Section 2, we summarize related work. In Section 3, we present a detailed deployment and protocol description for PaCoSIF. The optimization problem is formulated and solved in Section 4. In Section 5, we present the simulation results on PaCoSIF performance. Finally, we come to conclusions in Section 6.

## 2. Related Work

### 2.1. Spectrum Sensing with Full-Duplex Cognitive Radio

In half-duplex CRNs, spectrum sensing has to be scheduled sequentially with SU transmissions because the latter causes strong interference to the former. With the drastic progress toward full-duplex radio, it is feasible for SUs to carry out sensing and transmission simultaneously [8]. Most research on full-duplex sensing focuses upon the performance of single sensors. Cheng et al. [13] firstly introduce full-duplex sensing into single-channel non-time-slotted CRNs and then extend it into multi-channel CRNs [14]. Yang et al. [15] propose a new test statistics for a full-duplex auto-correlation detector. Ahmed et al. [16] investigate the achievable throughput gain of full-duplex sensing using directional antennas. Liao et al. [10] propose a novel listen-and-talk protocol for CRNs inspired by full-duplex sensing and solve the power control problem therein. Afifi et al. [9,11] investigate the optimal scheduling for full-duplex sensing and transmission in the context of single-channel and multi-channel overlay CRNs, respectively. As for underlay CRN, it has been found that full-duplex operation does not always enhance the throughput of SU [12], and an adaptive mode switching scheme is proposed therein. On the other hand, the performance of cooperative sensing among multiple full-duplex CRs has only been studied in [17], without considering the issue of spectrum heterogeneity. The optimal PU detection problem with full-duplex sensors in heterogeneous environments is investigated in this paper. The proposed solution is orthogonal to the performance of individual sensors and thus can be integrated with most current research on full-duplex sensing.

### 2.2. Cooperative Sensing in Spectrum-Heterogeneous Environments

Cooperative spectrum sensing exploits the diversity of multiple sensors to relax the sensitivity requirements on individual sensors or to mitigate the impact of multi-path fading and shadowing [23,25,26]. The sensing results of the cooperative sensors are fused in either a soft or hard manner, depending on whether the sensors involved report their raw samples or just binary decisions to the fusion center. So far, the optimal detection threshold has only been determined in the context of the soft fusion strategy and the likelihood-ratio test employed by an individual sensor [20,21,26,27,28,29,30,31]. However, the likelihood-ratio test requires a priori knowledge about the distribution of received signals, which is difficult to obtain in practice [32]. Furthermore, PaCoSIF adopts a hard fusion strategy rather than a soft one because the latter would cause significant interference to PU detection by transmitting large amounts of raw data.

As for hard decision fusion, the most common fusion rule is “K out of M” [23]. The OR rule adopted by PaCoSIF is a special case where K = 1. When cooperative sensors are deployed in spectrum-homogeneous environments, their missed detection rates are relaxed equally under the OR rule [24]. However, this solution is sub-optimal when SUs are located in heterogeneous environments and have different SINRs [25]. To solve this problem, Wu et al. [19] propose that an individual sensor should only fuse the decisions from other cooperative sensors that are not far away. Kim et al. [24] try to eliminate the heterogeneity by clustering the sensors into smaller groups according to their SINR. Particularly, the differences among the SINR of the sensors in the same group should be no larger than 1 dB. Peh et al. [25] attempt to mitigate the heterogeneity by excluding sensors with low SINR from data fusion. Compared with the above research, the novelty of PaCoSIF lies in the fact that spectrum heterogeneity is tackled by adaptive threshold adjustment rather than sensor clustering or exclusion. The optimization method proposed in PaCoSIF can be applied to a general heterogeneous CRN where the number of cooperative sensors is very limited.

The impact of transmission delay and bit error of the control channel upon the detection threshold setting has been investigated in [28,30]. In PaCoSIF, the instantaneous reverse channel acts as the control channel, and the transmission delay is negligible. The bit error of the control channel is not taken into consideration because only binary decisions modulated with pseudo-noise (PN) sequences are transmitted and the reception of PN sequences can be implemented rather reliably even when the SINR is low [33].

## 3. Deployment and Protocol Design of PaCoSIF

Figure 1 depicts a typical CRN deployment in spectrum heterogeneous environments. There exists only one PU, e.g., a digital TV transmitter, which occasionally transmits orthogonal-frequency-division- multiplexing (OFDM) signals. A cognitive radio network is deployed around the boundary of the so-called keep-out region. SUs access the vacant spectrum opportunistically when PU is idle. If the distance between an SU device and the PU transmitter is less than the keep-out radius *d*, SUs are required to ensure no interruption to the PU’s transmission. The CRN consists of multiple SUs equipped with full-duplex radio. At any time, only one SU link is scheduled to avoid mutual interference.

Even if the distance between the SU-Tx and PU is larger than *d*, the coverage area of the SU-Tx, i.e., the gray circle in Figure 1, may still overlap with the keep-out region. To avoid potential collisions with PU, the SU-Tx is usually required to detect PU signals within a much larger radius *D* in the context of non-cooperative sensing [24]. Alternatively, the SU-Tx in PaCoSIF collects the sensing results of the current SU-Rx via the reverse channel provided by full-duplex radio and then fuses them with local decisions to relax the sensitivity requirement on either sensor.

**Figure 2:**
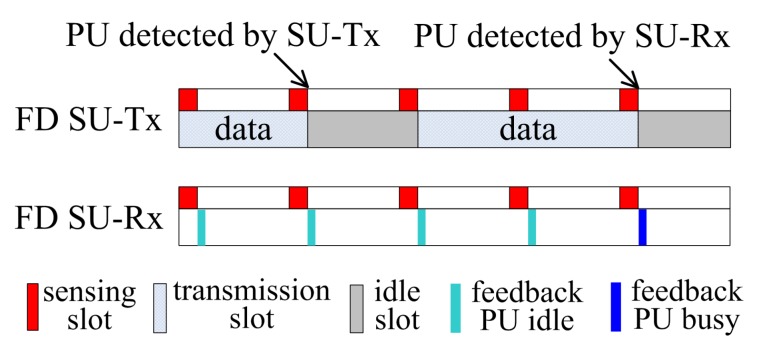
Scheduling of sensing, feedback and transmission in PaCoSIF.

The scheduling of data transmission, spectrum sensing, and decision feedback in PaCoSIF is described in Figure 2. A saturated traffic model is adopted for the CRN, i.e., the SU-Tx always has packets to send. The transmission of SU-Tx is time-slotted, and the sensing slot is scheduled just before each transmission slot. For the SU-Tx, simultaneous sensing and transmission is feasible owing to the self-interference suppression capability of full-duplex radio. The SU-Rx, on the other hand, has to distinguish PU signals from signals transmitted by the SU-Tx. At the end of each sensing slot, the SU-Rx reports its binary decision to the SU-Tx via the reverse channel. If the return of PU is detected by the SU-Tx or the SU-Rx, the SU-Tx abandons the transmission slot immediately.

We then turn to the protocol design of PaCoSIF. To coordinate the behavior of the SU-Tx and the SU-Rx, PaCoSIF assigns a unique signature, e.g., a pseudo-noise (PN) sequence, to each SU device as well as PU. The meanings of the signatures used in PaCoSIF are listed as follows:

- $PN(R_{i})$: the PN sequence assigned to the *i*-th SU-Rx indicates that a data transmission directed to it is to be initialized by the SU-Tx.
- PN(T): the PN sequence assigned to the SU-Tx indicates that PU is idle, and a data transmission to the intended receiver is allowed to be started.
- PN(U): the PN sequence assigned to PU indicates that PU is active, and current data transmission should be abandoned or suspended.

**Figure 3:**
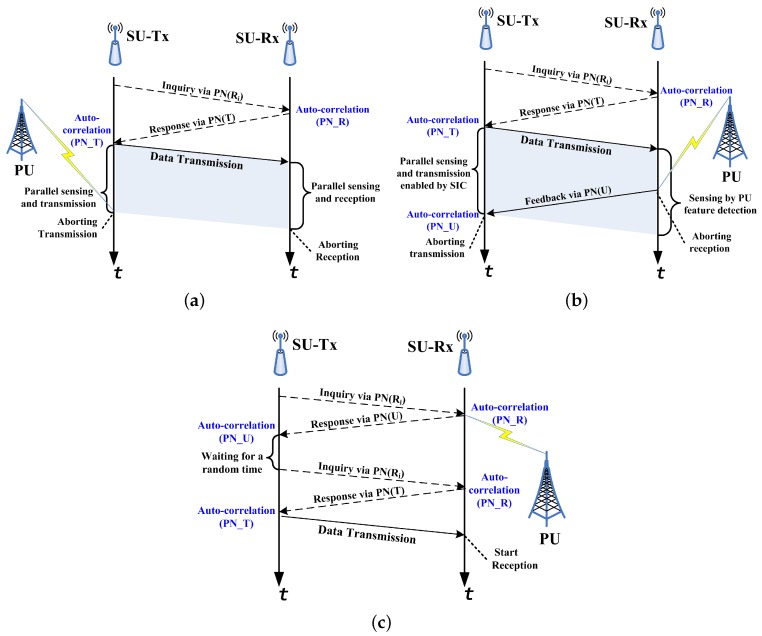
Flowcharts of the PaCoSIF. Three typical scenarios are represented: (**a**) PU’s return is detected by the SU-Tx; (**b**) PU’s return is detected by the SU-Tx; (**c**) multiple handshakes due to spectrum heterogeneity.

Three typical flowcharts illustrating how PaCoSIF works are presented in Figure 3. To ensure safe spectrum access, the SU-Tx firstly broadcasts the signature of the intended receiver before starting a new transmission, even if it does not detect the presence of PU signals. The low power-spectrum density of the PN sequence guarantees no interruption from the SU to the PU (The SU-Tx works in the underlay mode in this stage using the spectrum spreading waveform). Meanwhile, the intended SU-Rx can discern the signature by correlating the received signal with its own PN sequence; it then starts to sense the channel. If the feedback from the SU-Rx also indicates that PU is idle, the SU-Tx will start the data transmission in the overlay mode. During ongoing transmission, both the SU-Tx and the SU-Rx continue to detect PU signals. The SU-Tx employs an energy detector since the self-interference can be effectively eliminated by full-duplex radio, whereas the SU-Rx adopts an auto-correlating detector since it can reliably detect the cyclic prefix of the PU’s OFDM signals in spite of the strong interference led by SU transmissions [34]. If the SU-Tx detects the presence of PU, it will abandon the spectrum directly, as shown in Figure 3a. Otherwise, if the SU-Rx detects the return of PU, it will abort current reception and then start to transmit the signature of the PU, as shown in Figure 3b. The SU-Tx discerns the notification through correlation reception and then stops transmitting immediately.

Owing to spectrum heterogeneity, the presence of the PU may be detected only by the SU-Rx. In this case, after receiving the inquiry from the SU-Tx, the SU-Rx will send back the signature of the PU to suspend the transmission. The SU-Tx waits for a random amount of time before starting a new inquiry, as shown in Figure 3c. Until the PU is not detected by either sensor, the SU-Tx can access the spectrum safely. It is worth noting that this half-duplex handshake process is only necessary to initialize a new data transmission. For an established data transmission, the feedback from the SU-Rx will be delivered via the reverse channel of the full-duplex radio.

## 4. Optimizing Sensing Performance

In this section, we firstly formulate the sensing performance optimization problem in PaCoSIF, and then solve it using the Lagrange method. Furthermore, we propose a fast binary-searching algorithm to find the optimal solution numerically.

### 4.1. Problem Formulation

Spectrum sensing is the process by which SUs detect the presence of PUs by analyzing the sampled signals. It can be modeled as a binary hypothesis-testing problem, where $H_{1}$ represents PU is active and $H_{0}$ represents PU is idle. According to the signal processing method and test statistics, detectors can be divided into energy detectors [35], auto-correlation detectors [34], matched filter detectors [36], and others.

On one side, the SU-Tx in PaCoSIF carries out spectrum sensing and data transmission simultaneously owing to self-interference suppression enabled by the full-duplex radio. A common model for formulating the incoming signals of the SU-Tx is given as follows [9,10,17]:

(1) $$r_{tx}\left(n\right)=\left\{\begin{array}{c} w\left(n\right)+\chi s\left(n\right)\;\;\;\;H_{0}:\;\mathrm{PU}\;\mathrm{idle},\\ l\left(n\right)+w\left(n\right)+\chi s\left(n\right)\;H_{1}:\;\mathrm{PU}\;\mathrm{active}, \end{array}\right.$$

where w(n) represents channel noise, which is assumed to follow the zero-mean Gaussian distribution with variance $\sigma_{w}^{2}$, l(n) and s(n) are discrete samples of the signals transmitted by PU and the SU-Tx, respectively, which are assumed to be zero-mean random variables with variances $\sigma_{l}^{2}$ and $\sigma_{s}^{2}$, and χ is the factor that represents the degree of RSI. Only linear RSI is considered here for two reasons. Firstly, the impact of linear self-interference upon the performance of energy detectors has been well studied. Secondly, the threshold-optimization method of PaCoSIF does not depend upon specific detectors, and thus, it can be easily extended to incorporate non-linear self-interference.

When the energy detector is applied to the full-duplex SU-Tx, its performance in terms of the missed detection rate, $p_{tx}$, and the false alarm rate, $q_{tx}$, can be expressed as follows [9]:

(2) $$\begin{array}{cc} p_{tx} & =Q\left(-a_{1}\cdot \gamma_{tx}+b_{1}\right), \end{array}$$

(3) $$\begin{array}{cc} q_{tx} & =Q\left(c_{1}\cdot \gamma_{tx}-d_{1}\right), \end{array}$$

where Q(·) is the Q-function defined as $Q\left(x\right)=\frac{1}{\sqrt{2\pi }}\int_{x}^{\infty }exp\left(-\frac{u^{2}}{2}\right)du$, $\gamma_{tx}$ is the normalized detection threshold of the SU-Tx, $a_{1}=\sqrt{\frac{N}{2\alpha_{i}+2\alpha_{l}+2\alpha_{i}\alpha_{l}+1}}$, $b_{1}=\left(\alpha_{i}+\alpha_{l}+1\right)a_{1}$, $c_{1}=\sqrt{\frac{N}{2\alpha_{i}+1}}$, $d_{1}=\left(\alpha_{i}+1\right)c_{1}$, *N* is the number of samples taken within one sensing slot, $\alpha_{i}=\frac{\chi^{2}\sigma_{s}^{2}}{\sigma_{w}^{2}}$ is the power ratio between the RSI signal and channel noise, and $\alpha_{l}=\frac{\sigma_{l}^{2}}{\sigma_{w}^{2}}$ is the power ratio between the received PU signal and the channel noise. The SINR of the SU-Tx is defined as $\rho_{tx}=\frac{\sigma_{l}^{2}}{\sigma_{w}^{2}+\chi^{2}\sigma_{s}^{2}}$.

On the other side, the incoming signal of the SU-Rx can be modeled as follows:

(4) $$r_{rx}\left(n\right)=\left\{\begin{array}{c} w\left(n\right)+h\left(n\right)\;\;\;\;H_{0}:\;\mathrm{PU}\;\mathrm{idle},\\ l\left(n\right)+w\left(n\right)+h\left(n\right)\;H_{1}:\;\mathrm{PU}\;\mathrm{active}, \end{array}\right.$$

where h(n) and l(n) represent the discrete samples of signals from the SU-Tx and PU, respectively, which are assumed to be zero-mean random variables with variances $\sigma_{h}^{2}$ and $\sigma_{p}^{2}$, respectively (The power of PU signals received by the SU-Tx and SU-Rx might be different since they are deployed in heterogeneous environments. Therefore, we use a new symbol, $\sigma_{p}^{2}$, instead of $\sigma_{l}^{2}$, to emphasize the potential difference.).

Although there is no self-interference at the SU-Rx, the signal transmitted by the SU-Tx introduces a stronger interference, leading to a lower SINR for PU detection. There is rich literature on how to detect a weak signal from incoming signals [24,37]. In PaCoSIF, the SU-Rx adopts an auto-correlation detector that is capable of detecting PU signals with very low SINR. The detection performance in terms of the missed detection rate, $p_{rx}$, and the false alarm rate, $q_{rx}$, can be expressed as follows [34]:

(5) $$\begin{array}{cc} p_{rx} & =Q\left(-a_{2}\cdot \gamma_{rx}+b_{2}\right), \end{array}$$

(6) $$\begin{array}{cc} q_{rx} & =Q\left(c_{2}\cdot \gamma_{rx}\right), \end{array}$$

where $\gamma_{rx}$ is the normalized detection threshold of the SU-Rx, $c_{2}=\sqrt{\frac{T_{c}}{T_{c}+T_{d}}2N}$, $a_{2}=\frac{c_{2}}{1-v^{2}}$, $b_{2}=v\cdot a_{2}$, $v=\frac{\rho_{rx}}{1+\rho_{rx}}$, $\rho_{rx}=\frac{\sigma_{p}^{2}}{\sigma_{h}^{2}+\sigma_{w}^{2}}$, $T_{c}$ and $T_{d}$ are the numbers of cyclic prefix symbols and data symbols within an OFDM block.

The sensing results of the SU-Tx and the SU-Rx are fused using the OR rule. Let $P_{md}$ and $P_{fa}$ represent the missed detection rate and the false alarm rate of the fused decision, respectively. They can be represented as follows:

(7) $$\begin{array}{cc} P_{md} & =p_{tx}\cdot p_{rx}, \end{array}$$

(8) $$\begin{array}{cc} P_{fa} & =1-\left(1-q_{tx}\right)\left(1-q_{rx}\right). \end{array}$$

Given the bound on the missed detection rate, say $\beta_{0}$, $p_{tx}$ and $p_{rx}$ can be solved using Equation (7), and then, the thresholds $\gamma_{tx}$ and $\gamma_{rx}$ can be fixed using Equations (2) and (5). Finally, the false alarm rates $q_{tx}$, $q_{rx}$ and $P_{fa}$ can be deduced from Equations (3), (6) and (8), respectively. For conventional cooperative sensing mechanisms, the solution to Equation (7) is simply $p_{tx}=p_{rx}=\sqrt{\beta_{0}}$, i.e., the sensitivity of each sensor is relaxed equally. For example, if $\beta_{0}=0.1$, as proposed in the IEEE 802.22 standard [24], both $p_{tx}$ and $p_{rx}$ will be relaxed to 0.32. A larger missed detection rate leads to a smaller false alarm rate, and, thus, $P_{fa}$ is decreased via cooperation. However, the cooperative gain cannot be optimized if the sensors involved have different SINRs. In PaCoSIF, we adopt the NP criterion, and our goal is to minimize the false alarm rate given the bound on the missed detection rate by adjusting the thresholds of the two sensors. The optimization problem is represented as follows:

(9) $$\begin{array}{c} \underset{\gamma_{tx},\;\gamma_{rx}}{\arg\min}\;P_{fa}\\ s.\;t.\;\;P_{md}\leq \beta_{0}, \end{array}$$

where the bound on the missed detection rate is set as $\beta_{0}=0.1$ in this paper.

The notations used for the problem formulation and solution are listed in Table 1.

### 4.2. Problem Solution

To solve Equation (9), we firstly define the Lagrangian as

(10) $$F=P_{fa}+\lambda_{0}\left(P_{md}-\beta_{0}\right).$$

Using Equations (2)–(6), we obtain

(11) $$\begin{array}{cc} F & =Q\left(c_{1}\cdot \gamma_{tx}-d_{1}\right)+Q\left(c_{2}\cdot \gamma_{rx}\right)-Q\left(c_{1}\cdot \gamma_{tx}-d_{1}\right)\cdot Q\left(c_{2}\cdot \gamma_{rx}\right)\\ & +\lambda_{0}\left[Q\left(-a_{1}\cdot \gamma_{tx}+b_{1}\right)\cdot Q\left(-a_{2}\cdot \gamma_{rx}+b_{2}\right)-\beta_{0}\right]. \end{array}$$

Then, we try to find the optimal values of $\gamma_{tx}$ and $\gamma_{rx}$ by solving the following equations:

(12) $$\frac{\partial F}{\partial \gamma_{tx}}=\frac{\partial F}{\partial \gamma_{rx}}=\frac{\partial F}{\partial \lambda_{0}}=0.$$

Since $Q^{{}^{\prime }}\left(x\right)=-\frac{1}{\sqrt{2\pi }}\exp\left(-\frac{x^{2}}{2}\right)$, the solutions to Equation (12) can be expressed as

(13) $$\begin{array}{cc} \frac{1-Q(c_{2}\gamma_{rx})}{Q(b_{2}-a_{2}\gamma_{rx})} & =\frac{\lambda_{0}b_{1}}{c_{1}}\exp\left[\frac{1}{2}\left({\left(c_{1}\gamma_{tx}-d_{1}\right)}^{2}-{\left(b_{1}-a_{1}\gamma_{tx}\right)}^{2}\right)\right], \end{array}$$

(14) $$\begin{array}{cc} \frac{1-Q(c_{1}\gamma_{tx}-d_{1})}{Q(b_{1}-a_{1}\gamma_{tx})} & =\frac{\lambda_{0}b_{2}}{c_{2}}\exp\left[\frac{1}{2}\left({\left(c_{2}\gamma_{rx}\right)}^{2}-{\left(b_{2}-a_{2}\gamma_{rx}\right)}^{2}\right)\right], \end{array}$$

(15) $$\begin{array}{cc} \beta_{0} & =Q\left(b_{1}-a_{1}\gamma_{tx}\right)\cdot Q\left(b_{2}-a_{2}\gamma_{rx}\right). \end{array}$$

**Figure 4:**
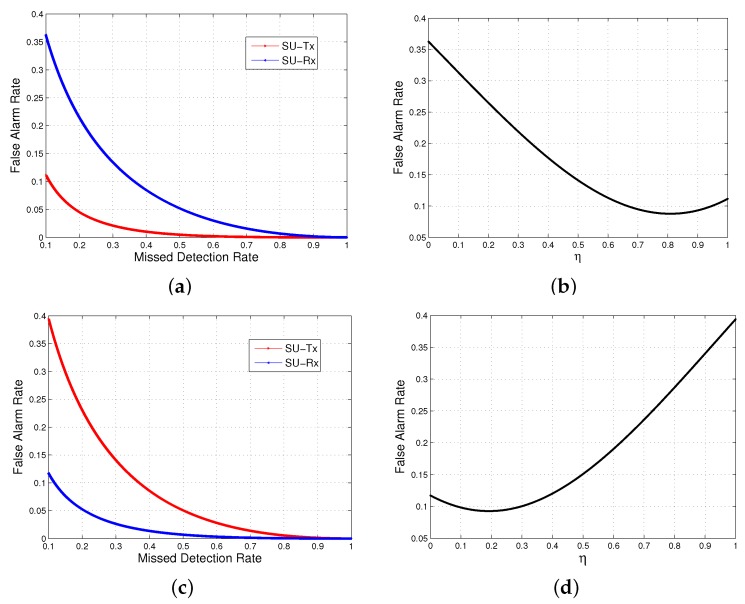
The false alarm rates of an individual sensor versus cooperative sensing. (**a**) ROC of an individual sensor ($\rho_{tx}>\rho_{rx}$); (**b**) false alarm rate of sensing fusion ($\rho_{tx}>\rho_{rx}$); (**c**) ROC of an individual sensor ($\rho_{tx}<\rho_{rx}$); (**d**) false alarm rate of sensing fusion ($\rho_{tx}<\rho_{rx}$).

However, Equations (13)–(15) are not analytically tractable. To obtain a numerical solution for $P_{fa}$, we change the intermediate variables from $\gamma_{tx}$ and $\gamma_{rx}$ to $p_{tx}$ and $p_{rx}$ respectively. Combining Equations (2)–(6), we can express the false alarm rate of each detector in terms of its missed detection rate:

(16) $$\begin{array}{c} q_{tx}=Q\left(-m_{1}\cdot Q^{-1}\left(p_{tx}\right)+u_{1}\right), \end{array}$$

(17) $$\begin{array}{c} q_{rx}=Q\left(-m_{2}\cdot Q^{-1}\left(p_{rx}\right)+u_{2}\right), \end{array}$$

where $m_{1}=\sqrt{\left(1+\frac{2\alpha_{l}\left(1+\alpha_{i}\right)}{1+2\alpha_{i}}\right)}$, $u_{1}=\alpha_{l}\sqrt{\frac{N}{1+2\alpha_{i}}}$, $m_{2}=1-\rho^{2}$, $u_{2}=\rho \sqrt{\frac{2NT_{c}}{T_{c}+T_{d}}}$. Equations (16) and (17) define the receiver-operating curves (ROC) of the SU-Tx and SU-Rx, respectively. When the SINR of the two sensors differs significantly, their ROC curves also exhibit different properties, as shown in Figure 4a,c. The ROC of the sensor with a higher SINR always lies beneath that of the sensor with a lower SINR. Therefore, relaxing the missed detection rate of the sensor with a lower SINR leads to a more significant decrease of the false alarm rate. A concrete example is provided in Table 2. The cooperative gain (CG) is defined as the logarithmic ratio between the false alarm rate of non-cooperative sensing (e.g., FDNCS) and that of cooperation sensing (e.g., PaCoSIF or FDCS), i.e., $\mathrm{CG}=log\left(\frac{P_{fa}^{NCS}}{P_{fa}^{CS}}{|}_{P_{md}=\beta_{0}}\right)$. From Table 2, we can find that more cooperative gain can be harvested in heterogeneous environments by assigning a larger missed detection rate to the sensor with a lower SINR. Inspired by this intuition, PaCoSIF adaptively adjusts the missed detection rates of the two sensors in accordance with their SINR to minimize $P_{fa}$.

Let $p_{tx}=\beta_{0}^{\eta }$ and $p_{rx}=\beta_{0}^{1-\eta }$, where η and 1-η are the exponents assigned to the SU-Tx and the SU-Rx, respectively, for relaxing the missed detection rate. Then, the optimization problem in Equation (9) can be rewritten as follows:

$$\begin{array}{c} \underset{\eta }{\arg\min}\;\left(q_{tx}+q_{rx}-q_{tx}\cdot q_{rx}\right)\\ \;s.\;t.\;\;0<\eta <1. \end{array}$$

Figure 4b,d represent the relationship between $P_{fa}$ and η in the scenarios of Figure 4a,c, respectively. The optimal solution, $\eta^{*}$, for Equation (18) is obtained at the point where the derivative of $P_{fa}$ is zero. As for the properties of the derivative, we have the following theorem.

**Theorem** **1.** The derivative $\frac{dP_{fa}}{d\eta }$ is monotonous in sign within the interval (0,1), and there exists one and only one value, $\eta^{*}$, which makes the derivative zero.

**Proof.**  The derivative of $P_{fa}$ relative to η is

(18) $$\begin{array}{c} \frac{dP_{fa}}{d\eta }=\frac{dP_{fa}}{dp_{tx}}\cdot \frac{dp_{tx}}{d\eta }=\left(\beta_{0}^{\eta }\ln\beta_{0}\right)\left[\frac{dq_{tx}}{dp_{tx}}\left(1-q_{rx}\right)+\frac{dq_{rx}}{dp_{rx}}\cdot \frac{dp_{rx}}{dp_{tx}}\left(1-q_{rx}\right)\right]\\ \;=\ln\beta_{0}\left[\beta_{0}^{1-\eta }\left(1-q_{tx}\left(\beta_{0}^{\eta }\right)\right)|\frac{dq_{rx}}{dp_{rx}}{{|}_{p_{rx}=\beta_{0}^{1-\eta }}|-\beta_{0}^{\eta }\left(1-q_{rx}\left(\beta_{0}^{1-\eta }\right)\right)|\frac{dq_{tx}}{dp_{tx}}|}_{p_{tx}=\beta_{0}^{\eta }}|\right]. \end{array}$$

To obtain the expressions for $\frac{dq_{tx}}{dp_{tx}}$ and $\frac{dq_{rx}}{dp_{rx}}$, we firstly denote $q_{k}=Q\left(s_{k}\right)$, where $s_{k}=-m_{k}y_{k}+u_{k}$, $y_{k}=Q^{-1}\left(p_{k}\right)$, k=1 refers to the SU-Tx and k=2 refers to the SU-Rx. Then, the derivative of $q_{k}$ can be represented as follows:

(19) $$\begin{array}{c} \frac{dq_{k}}{dp_{k}}=\frac{dq_{k}}{ds_{k}}\cdot \frac{ds_{k}}{dp_{k}}=\frac{m_{k}}{\sqrt{2\pi }}\exp\left(-\frac{s_{k}^{2}}{2}\right)\frac{dy_{k}}{dp_{k}}\\ \;=-m_{k}\exp\left(\frac{y_{k}^{2}-s_{k}^{2}}{2}\right)<0. \end{array}$$

Therefore, $q_{k}$ is a monotonously decreasing function, which has already been revealed by the ROC in Figure 4a,c.

Now, we deduce the properties of $\frac{dq_{k}}{dp_{k}}$. Let us write $f\left(y_{k}\right)=y_{k}^{2}-s_{k}^{2}=\left(1-m_{k}^{2}\right)y_{k}^{2}+2m_{k}u_{k}y_{k}-u_{k}^{2}$. For the SU-Tx, $1-m_{1}^{2}<0$, the parabola $f(y_{1})$ opens downward and its axis of symmetry is

(20) $$y_{1}^{*}=\frac{m_{1}u_{1}}{m_{1}^{2}-1}=\frac{\sqrt{N(1+2\alpha_{i}+2\alpha_{l}+2\alpha_{i}\alpha_{l})}}{2(1+\alpha_{i})}\approx \frac{\sqrt{N}}{2}>0.$$

Since $\beta_{0}<p_{1}<1$, we have $y_{1}<Q^{-1}\left(\beta_{0}\right)=1.282$. Meanwhile, *N* is on the order of several hundreds or thousands, so we further have $y_{1}<y_{1}^{*}$. Therefore, $f(y_{1})$ is a monotonously increasing function. When $\eta \to 0^{+}$, we have $p_{1}\to 1$, $y_{1}\to -\infty$, $f(y_{1})\to -\infty$ and finally conclude that $\frac{dq_{1}}{dp_{1}}{|}_{\eta \to 0^{+}}\to 0$.

For the SU-Rx, $1-m_{2}^{2}>0$, the parabola $f(y_{2})$ opens upward. Its axis of symmetry is

(21) $$y_{2}^{*}=\frac{m_{2}u_{2}}{m_{2}^{2}-1}=-\frac{1-\rho^{2}}{\rho (2-\rho^{2})}\sqrt{\frac{2NT_{c}}{T_{c}+T_{d}}}\approx -\frac{1}{\rho }\sqrt{\frac{NT_{c}}{2(T_{c}+T_{d})}}<0.$$

The sign of $y_{2}^{*}$ is negative, and its absolute value is large. Meanwhile, $p_{2}$ approaches 1 rapidly as $y_{2}$ decreases—for example, when $y_{2}=-20$, $1-p_{2}<10^{-88}$. Therefore, we only consider the practical interval of $y_{2}>y_{2}^{*}$, within which $f(y_{2})$ is a monotonously increasing function. When $\eta \to 1^{-}$, we  have $p_{2}\to 1$, $y_{2}\to -\infty$ and conclude that $\frac{dq_{2}}{dp_{2}}{|}_{\eta \to 1^{-}}\to 0$.

Substituting the limit of $\frac{dq_{k}}{dp_{k}}$ into Equation (18), we have

(22) $$\begin{array}{c} \frac{dP_{fa}}{d\eta }{|}_{\eta \to 0^{+}}=\beta_{0}\ln\beta_{0}|\frac{dq_{2}}{dp_{2}}{|}_{p_{2}=\beta_{0}}|<0, \end{array}$$

and

(23) $$\begin{array}{c} \frac{dP_{fa}}{d\eta }{|}_{\eta \to 1^{-}}=-\beta_{0}\ln\beta_{0}|\frac{dq_{1}}{dp_{1}}{|}_{p_{1}=\beta_{0}}|>0. \end{array}$$

Since $\frac{dP_{fa}}{d\eta }$ is continuous within the interval (0,1), there must exist one point $\eta^{*}$ that satisfies $\frac{dP_{fa}}{d\eta }=0$.

Finally, we discuss the sign of $\frac{dP_{fa}}{d\eta }{|}_{\eta =0.5}$. Since

(24) $$\frac{dP_{fa}}{d\eta }{|}_{\eta =0.5}=\left(\sqrt{\beta_{0}}\ln\beta_{0}\right)\left[\left(1-q_{1}\left(\sqrt{\beta_{0}}\right)\right)|q_{2}^{{}^{\prime }}\left(\sqrt{\beta_{0}}\right)|-\left(1-q_{2}\left(\sqrt{\beta_{0}}\right)\right)|q_{1}^{{}^{\prime }}\left(\sqrt{\beta_{0}}\right)|\right],$$

the sign of $\frac{dP_{fa}}{d\eta }{|}_{\eta =0.5}$ depends upon the ROCs of both the SU-Tx and the SU-Rx. The following two cases will be discussed separately.

Case I: The SU-Tx outperforms the SU-Rx.

In this case, the ROC of the SU-Tx lies beneath that of the SU-Rx, so we have

(25) $$|q_{1}^{{}^{\prime }}\left(\sqrt{\beta_{0}}\right)|<|q_{2}^{{}^{\prime }}\left(\sqrt{\beta_{0}}\right)|,$$

and

(26) $$q_{1}\left(\sqrt{\beta_{0}}\right)<q_{2}\left(\sqrt{\beta_{0}}\right)<1.$$

Substituting inequalities (25) and (26) into Equation (24), we have $\frac{dP_{fa}}{d\eta }{|}_{\eta =0.5}<0$. The monotonicity of $\frac{dq_{k}}{dp_{k}}$ ensures the following results: (i) $\frac{dP_{fa}}{d\eta }$ is always negative when 0<η<0.5, and (ii) there is only one point, $\eta^{*}$, located within the interval (0.5,1) that makes the derivative of $P_{fa}$ zero.

Case II: The SU-Rx outperforms the SU-Tx.

In this case, the ROC of the SU-Rx lies beneath that of the SU-Tx. Similarly, we can deduce that $\frac{dP_{fa}}{d\eta }{|}_{\eta =0.5}>0$. Therefore, $\frac{dP_{fa}}{d\eta }$ is positive when 0.5<η<1, and the unique point, $\eta^{*}$, which makes the derivative of $P_{fa}$ zero be within the interval (0,0.5). ☐

Since the derivative of $P_{fa}$ is monotonous in sign, the value $\eta^{*}$ that makes the derivative zero can be found using a standard binary-searching algorithm. This is presented in Algorithm 1, where $\eta_{s}$ and $\eta_{e}$ represent the start and end points of the current search interval, respectively, ϵ is the predefined convergence threshold. Initially, the searching interval is set as (0,0.5) or (0.5,1) depending on the SINR difference between the SU-Tx and SU-Rx. After each iteration, the search interval is cut in half, and $\eta^{*}$ is further searched within the sub-interval satisfying the condition that the signs of $\frac{dP_{fa}}{d\eta }|\eta_{s}$ and $\frac{dP_{fa}}{d\eta }|\eta_{e}$ are different. The accuracy of $\eta^{*}$ converges to 1/n rapidly after $O(\log_{2}n)$ iterations.

**Algorithm 1** Binary searching algorithm to find $\eta^{*}$
**Intput:**   Parameters of detector at SU-Tx: $\alpha_{l}$, $\alpha_{i}$, *N*;    Parameters of detector at SU-Rx: ρ, *N*, $T_{c}$, $T_{d}$;    The upper bound on the missed detection rate: $\beta_{0}$. **Output:**   The optimal exponent assigned to the SU-Tx for sensitivity relaxation: $\eta^{*}$. 1: **if**
$q_{tx}\left(\beta_{0}\right)$ = $q_{rx}\left(\beta_{0}\right)$
**then** 2:     $\eta^{*}$ = 0.5 3: **else**
**if**
$q_{tx}\left(\beta_{0}\right)$ < $q_{rx}\left(\beta_{0}\right)$
**then** 4:     $\eta_{s}$ = 0.5, $\eta_{e}$ = 1 5: **else** 6:     $\eta_{s}$ = 0, $\eta_{e}$ = 0.5 7: **end if** 8: **while**
$|P_{fa}^{{}^{\prime }}\left(\eta_{s}\right)-P_{fa}^{{}^{\prime }}\left(\eta_{e}\right)|>\epsilon$
**do** 9:     $\eta_{i}=\left(\eta_{s}+\eta_{e}\right)/2$ 10:    **if**
$P_{fa}^{{}^{\prime }}\left(\eta_{i}\right)$ has the same sign with $P_{fa}^{{}^{\prime }}\left(\eta_{s}\right)$
**then** 11:           $\eta_{s}=\eta_{i}$ 12:     **else** 13:           $\eta_{e}=\eta_{i}$ 14:     **end if** 15: **end while** 16: $\eta^{*}=\left(\eta_{s}+\eta_{e}\right)/2$


## 5. Performance Evaluation

In this section, we provide simulation results to evaluate the performance of PaCoSIF against that of conventional full-duplex cooperative sensing (FDCS) [17] and full-duplex non-cooperative sensing (FDNCS) [9]. In particular, we focus upon the performance enhancement obtained through threshold optimization in PaCoSIF.

### 5.1. Sensing Performance

The sensing performance is evaluated with the false alarm rate when the same bound, $\beta_{0}=0.1$, is given on the missed detection rate. The PU signal propagation model is borrowed from the IEEE 802.22 standard [24]. The power of the received PU signal at a distance of *r* is $E_{t}\cdot r^{-\alpha }$, where $E_{t}=64\;\mathrm{dBm}$ is the PU transmission power, and α=3.5 is the attenuation coefficient. The number of data symbols in one OFDM block of PU signals is $T_{d}=32$, and the number of cyclic-prefix symbols is $T_{c}=8$[34]. The SU-Tx is statically deployed near the border of the keep-out region and the power of its received PU signals is $\sigma_{l}^{2}=-100\;\mathrm{dBm}$. On the contrary, the SU-Rx is randomly located around the SU-Tx and its SINR varies. The power of the SU-Tx signals received by the SU-Rx is $\sigma_{h}^{2}=-80\;\mathrm{dBm}$ and the powers of both the channel noise and the RSI are $\sigma_{w}^{2}=-90\;\mathrm{dBm}$ (The impact of RSI upon the performance of a full-duplex sensor has been well investigated in [9,10] and is out of the scope of this paper. Instead, we focus upon the impact of spectrum heterogeneity and assume that the power of RSI is on the same level as the channel noise, which has been shown to be feasible with prototype experiments [7].). The SINR of the SU-Tx is a constant of $\rho_{tx}=$−13 dB, whereas the SINR of the SU-Rx $\rho_{rx}$ varies within the interval [-3dB,-23dB]. The SINR difference, $\Delta \rho =\rho_{rx}-\rho_{tx}$, is used as the metric of spectrum heterogeneity.

An energy detector is applied to the SU-Tx in all of the three sensing mechanisms, and an auto-correlation detector is employed by the SU-Rx in PaCoSIF and FDCS. We also align the performance of the single detector to emphasize the effect of threshold optimization. The numbers of samples taken by the SU-Tx and the SU-Rx during one sensing slot are $N_{tx}=2100$ and $N_{rx}=7000,$ respectively. Under such a parameter setting, the sensing performance of SU-Rx is similar to that of SU-Tx when they are deployed in the vicinity.

#### 5.1.1. Sensing Performance with Perfect SINR Information

**Figure 5:**
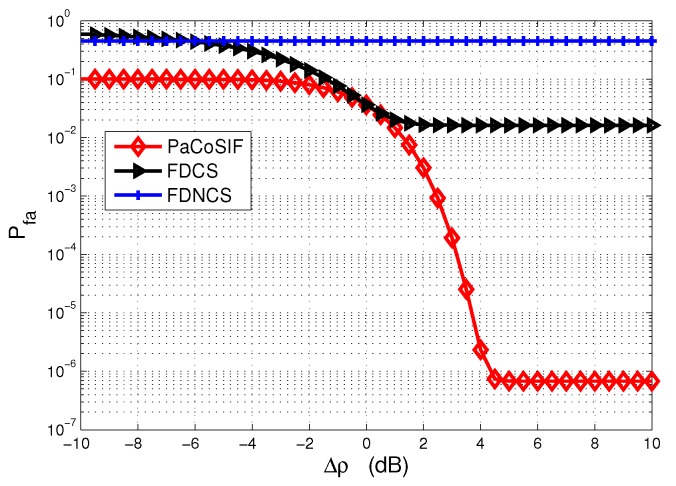
The false alarm rate of the fused decisions in spectrum-heterogeneous environments.

**Figure 6:**
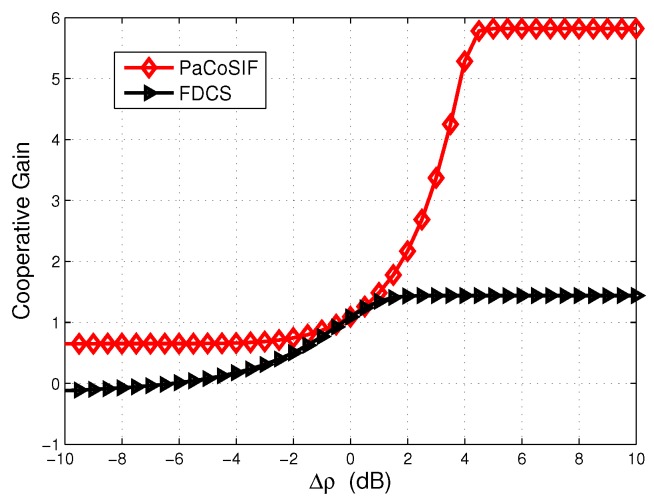
Cooperative gain in spectrum-heterogeneous environments.

We first assume that the SINR information is perfect and conduct numerical simulations to study the sensing performance of PaCoSIF, FDCS, and FDNCS in spectrum-heterogeneous environments. The simulation results are shown in Figure 5. For FDNCS, the false alarm rate remains constant as Δρ changes, since sensing is carried out by the SU-Tx itself. On the contrary, the false alarm rates of both PaCoSIF and FDCS decrease as the SINR of SU-Rx becomes higher, and, consequently, the cooperative gain is increased, as shown in Figure 6.

**Figure 7:**
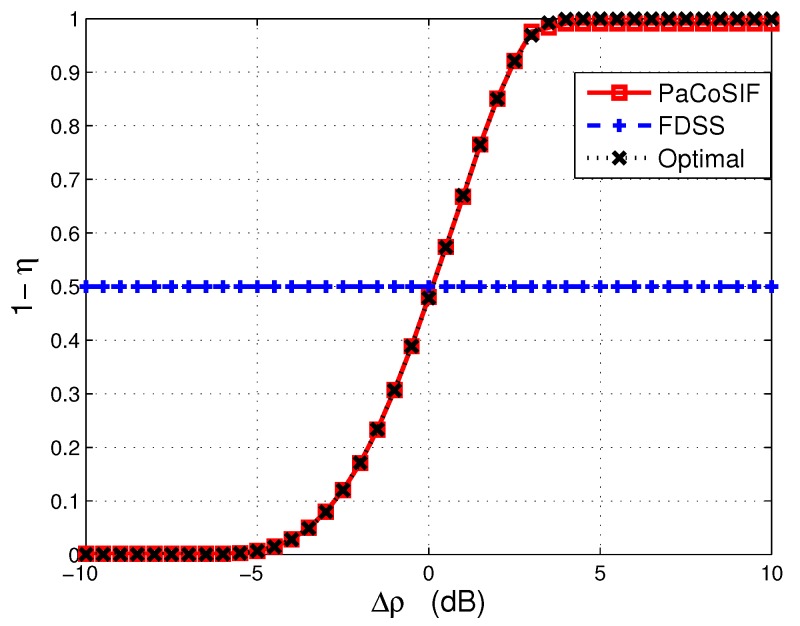
The exponent assigned to the SU-Rx in spectrum-heterogeneous environments.

When the SINR of SU-Rx is high, its detector outperforms that of the SU-Tx, and, therefore, the feedback from the SU-Rx would drastically improve the link-level sensing performance. In such a case, PaCoSIF tries to make full use of the feedback from the SU-Rx by assigning a small missed detection rate to the SU-Rx. For example, when Δρ=10dB, the missed detection rate of the SU-Rx is $p_{rx}\approx 0.1$, and the exponent assigned to the SU-Rx is 1-η≈1, as shown in Figure 7. However, the false alarm rate of the SU-Rx is still low owing to its high SINR. Meanwhile, the exponent assigned to the SU-Tx is η≈0, and a low false alarm rate is obtained since its missed detection rate, $p_{tx}$, has been relaxed to be sufficiently large. On the contrary, the missed detection rates of the two sensors in FDCS are relaxed with the same exponent 0.5. As a result, the cooperative gain of PaCoSIF is four orders higher than that of the FDCS.

When the SINR of the SU-Rx decreases below that of the SU-Tx, i.e., Δρ<0dB, feedback from the SU-Rx would “contaminate” the fused decision rather than improve it. As a result, the false alarm rate of the FDCS may be even larger than that of the FDNCS when the SINR of SU-Rx is extremely low and the cooperative gain in this scenario is negative. On the contrary, PaCoSIF tries to mitigate the harmful impact of the SU-Rx by sufficiently relaxing its missed detection rate. For example, when Δρ=-10dB, the exponent assigned to the SU-Rx is 1-η≈0, as shown in Figure 7. Consequently, the missed detection rate of the SU-Rx is $p_{rx}\approx 1$, and its false alarm rate is limited to a relatively small value. The adaptive sensitivity relaxation of PaCoSIF ensures that the cooperative gain is always positive.

From Figure 7, we also observe that the exponent η solved with Algorithm 1 is always close to the optimal solution, $\eta^{*}$, of Equation (18), which is obtained through exhaustive-searching. Meanwhile, the binary-searching algorithm presented in Algorithm 1 takes far less time than the exhaustive-searching method.

#### 5.1.2. Sensing Performance with Imperfect SINR Information

The performance of the optimization algorithm in PaCoSIF depends upon the accuracy of the SINR information. However, owing to limited samples taken within one sensing slot, there is a certain extent of uncertainty in the estimated power of the channel noise, leading to SINR estimation error [38]. In this section, we study the sensing performance with imperfect SINR information using Monte Carlo simulations.

When the variance of channel noise is $\sigma^{2}$, we assume that the estimation is distributed uniformly within the interval $\left[\frac{1}{\mu }\sigma^{2},\mu \sigma^{2}\right]$, where $\mu =10^{x/10}$, and *x* is the noise uncertainty in dB. Other parameters of the sensors and the signal propagation model are kept the same. For each pair of Δρ and *x*, we run $10^{5}$ Monte Carlo simulations and analyze the distribution of the false alarm rate $P_{fa}$.

**Figure 8:**
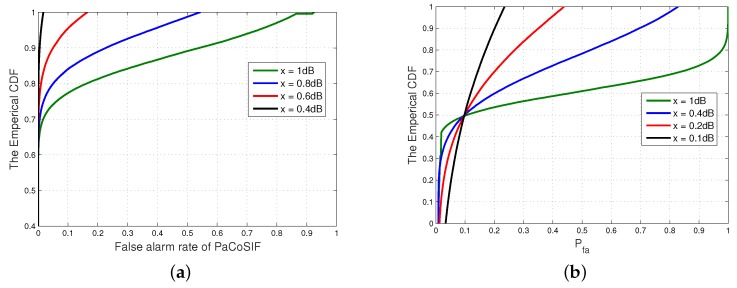
The distribution of the false alarm rate under different noise uncertainties. (**a**) Δρ=4dB; (**b**) Δρ=-4dB.

Figure 8 shows the distributions of $P_{fa}$ when Δρ is 4 dB and –4 dB. Without SINR uncertainty, i.e., x=0dB, the optimal false alarm rates are $2.34\times 10^{-6}$ and 0.098, respectively. For each Δρ, the noise uncertainty *x* varies between 0 dB and 1 dB. As the SINR uncertainty increases, the distribution range of $P_{fa}$ enlarges significantly. For example, when noise uncertainty is x=1dB, the largest $P_{fa}$ will approach 1. SINR estimation error degrades the performance of PaCoSIF in two ways. Firstly, the detection threshold of an individual sensor becomes inaccurate. Secondly, the assigned exponent η becomes sub-optimal.

**Figure 9:**
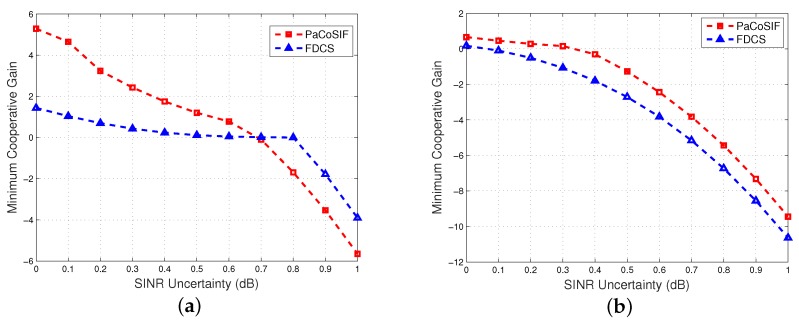
The impact of noise uncertainty upon the minimum cooperative gain. (**a**) Δρ=4dB; (**b**) Δρ=-4dB.

Now, we investigate the worst and average sensing performance of PaCoSIF as the SINR uncertainty increases. Figure 9 shows the relationship between the minimum cooperative gain and the SINR uncertainty when Δρ is 4 dB and −4 dB, respectively. The minimum cooperative gains of both PaCoSIF and FDCS decrease drastically as SINR uncertainty enlarges. In the worst case, the cooperative gain of PaCoSIF may be even smaller than that of FDCS since SINR error misleads the optimization process of PaCoSIF.

**Figure 10:**
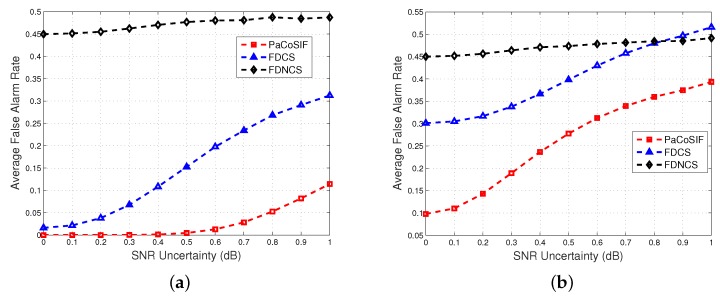
The impact of noise uncertainty upon the mean false alarm rate. (**a**) Δρ=4dB; (**b**) Δρ=-4dB.

Figure 10 shows the average values of $P_{fa}$ of PaCoSIF, FDCS and FDNCS with respect to the SINR uncertainty. It is not surprising to find that the average false alarm rates of all three mechanisms increase as noise uncertainty grows. When the SINR of SU-Rx is low and the SINR uncertainty is large, e.g., Δρ=-4dB and x>0.8dB, the average false alarm rate of FDCS becomes larger than that of FDNCS. On the contrary, the average false alarm rate of PaCoSIF is more robust against noise uncertainty and is always smaller than that of FDNCS when x<1dB.

### 5.2. CRN Throughput Enhancement

In this section, we study the CRN throughput of PaCoSIF through Monte Carlo simulations and compare it to those of FDCS and FDNCS. The CRN throughput is related with SU’s sensing performance as well as PU’s activity pattern. Here, we simulate the data flow of the PU with a frame-based ON–OFF model, where an ON period represents the duration within which the PU is transmitting frames and an OFF period represents the duration within which PU is idle [24]. The lengths of ON or OFF durations obey the exponential distribution. The mean ON period is 100 ms, and the payload of PU is 0.4. Each frame of PU lasts for 10 ms. The SU’s data flow is slot based. The length of one slot is 1 ms. A successful CRN transmission slot is the one within which PU is idle and no false alarm is reported by either SU-Tx or SU-Rx. Each Monte Carlo simulation lasts for 100 s, i.e., $10^{5}$ slots. As for SU transmission, the bandwidth is 6 MHz and the SINR is 10 dB.

#### 5.2.1. CRN Throughput with Perfect SINR Information

**Figure 11:**
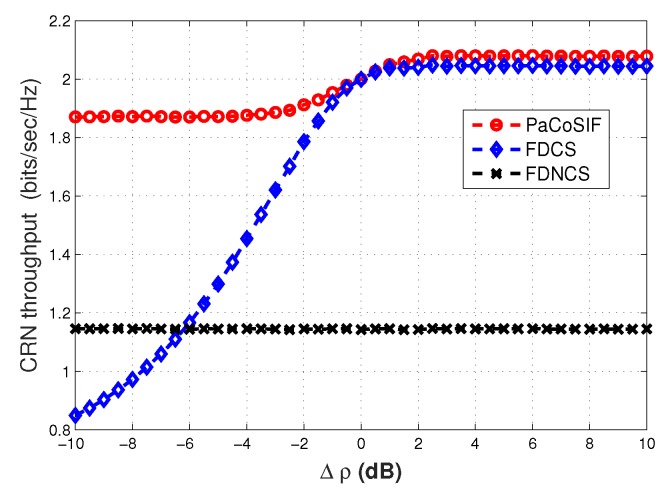
CRN throughput in spectrum-heterogeneous environments.

We carry out 500 Monte Carlo simulations with perfect SINR information to study the CRN throughput in spectrum-heterogeneous environments. The average CRN throughput with respect to the SINR difference Δρ is shown in Figure 11.

Since the false alarm rates of both PaCoSIF and FDCS decrease with the growth of Δρ, we are not surprised to see that the corresponding CRN throughput is boosted as Δρ becomes larger. When Δρ=0dB in spectrum-homogeneous environments, the throughput of PaCoSIF is just the same as that of FDCS. Otherwise, PaCoSIF always outperforms FDCS. By comparison, the CRN throughput of FDNCS does not change as Δρ varies, since the SU-Rx is not involved in sensing. When the SINR of SU-Rx is extremely low, e.g., Δρ<-6dB, the CRN throughput of FDCS is lower than that of FDNCS because the harmful feedback from the SU-Rx increases the false alarm rate of fused decision.

The difference between the changing rate of CRN throughput in Figure 11 and that of the false alarm rate in Figure 5 is worth noting. When Δρ grows from 0 dB to 10 dB, the false alarm rate of PaCoSIF drops by four orders lower than that of FDCS, but the absolute value of the decrease in $P_{fa}$ is actually small. Therefore, the throughput enhancement of PaCoSIF against FDCS in this scenario is not that significant. On the contrary, when Δρ is reduced from 0 dB to −10 dB, the false alarm rate of PaCoSIF is only four times lower than that of FDCS, but the absolute value of the increase in $P_{fa}$ is large. Therefore, the throughput enhancement of PaCoSIF against FDCS in this extreme scenario is significant.

#### 5.2.2. CRN Throughput with Imperfect SINR Information

**Figure 12:**
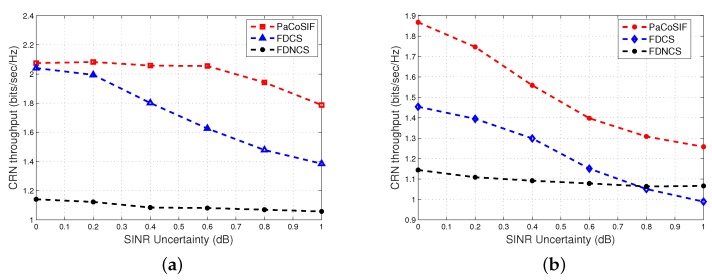
The impact of noise uncertainty upon the CRN throughput. (**a**) Δρ=4dB; (**b**) Δρ=-4dB.

Finally, we carry out Monte Carlo simulations to study the impact of SINR error upon the CRN throughput. The parameters of sensors as well as traffic model of PU and SU are the same as in the previous section. We also consider Δρ=4dB and Δρ=-4dB as examples. The CRN throughputs of PaCoSIF, FDCS, and FDNCS with respect to noise uncertainty are shown in Figure 12.

The throughput of CRN mainly depends upon $P_{fa}$. The average false alarm rates of PaCoSIF and FDCS are sensitive to SINR uncertainty, as shown in Figure 10. As a result, the CRN throughputs of PaCoSIF and FDCS decrease drastically as the SINR uncertainty *x* increases. By contrast, the CRN throughput of FDNCS is less sensitive to SINR uncertainty. Therefore, the throughput enhancement of PaCoSIF against FDNCS will diminish as *x* grows. Under some specific conditions, e.g., Δρ=-4dB and x>0.8dB, the CRN throughput of FDCS can even be lower than that of FDNCS.

## 6. Conclusions

Spectrum sensing using full-duplex CRs is a promising way to enhance spectrum awareness and efficiency. However, the issue of full-duplex sensing in spectrum-heterogeneous environments has not been addressed. In this paper, we propose a light-weight cooperative sensing framework called PaCoSIF for full-duplex CRN deployed in heterogeneous environments. In PaCoSIF, only a communicating SU pair consisting of an SU-Tx and an SU-Rx is involved in cooperation. The former acts as the fusion center and collects the hard decision of the SU-Rx periodically via the reverse channel. The sensing results are then fused using the OR rule. Since the cooperation is limited in an SU pair, a dedicated control channel is not required. More importantly, the sensitivity requirements of each cooperative sensor are relaxed in accordance with the SINR information. Optimal detection thresholds under the NP criterion are obtained using the Lagrange method and further solved numerically with a fast binary-searching algorithm. Simulation results show that, with perfect SINR information, the PaCoSIF can achieve optimal sensing performance. When the full-duplex CRN is deployed in spectrum-heterogeneous environments, the cooperative gain in the sensing performance and the CRN throughput will be boosted significantly against the conventional cooperative sensing mechanism. Our work also reveals that cooperation among sensors in spectrum-heterogeneous environments does not always lead to an enhanced performance unless adaptive sensitivity relaxation is adopted.

The PaCoSIF protocol design has only taken the point-to-point SU link into consideration. We are working on extending it to incorporate multiple SU links. Furthermore, the impact of spatial correlation among SU channels will also be considered.

## Figures and Tables

**Table 1:** Notations used for problem formulation and solution.

Symbol	Interpretation	Comment
$P_{md}$	The link level missed detection rate	
$P_{fa}$	The link level false alarm rate	
$\beta_{0}$	The bound on the missed detection rate of the CRN	A typical value is 0.1 as proposed in the IEEE 802.22 standard
*p*	The missed detection rate of the SU-Tx or SU-Rx	
*q*	The false alarm rate of the SU-Tx or SU-Rx	
γ	The detection threshold of the SU-Tx or SU-Rx	
Δρ	The difference of the SINR between the SU-Rx and the SU-Tx, i.e., $\rho_{rx}-\rho_{tx}$	Used as an indicator for spectrum heterogeneity
η	The exponent assigned to the SU-Tx for relaxing the missed detection rate	The exponent assigned to the SU-Rx is 1-η

**Table 2:** Case studies for sensitivity relaxation in FDNCS, FDCS, and PaCoSIF.

Sensing Mechanism	Δρ= 0 dB				Δρ= 3 dB			
	$p_{\mathrm{tx}}$	$p_{\mathrm{rx}}$	$P_{\mathrm{fa}}$	CG	$p_{\mathrm{tx}}$	$p_{\mathrm{rx}}$	$P_{\mathrm{fa}}$	CG
FDNCS	0.1	N/A	0.1	N/A	0.1	N/A	0.1	N/A
FDCS	0.316	0.316	0.0365	0.438	0.316	0.316	0.017	0.770
PaCoSIF	0.316	0.316	0.0365	0.438	0.945	0.106	$1.94\times 10^{-4}$	2.712

## Abbreviations

The following abbreviations are used in this manuscript:

PaCoSIF	Pairwise Cooperative Sensing with Instantaneous Feedback
FDNCS	Full-Duplex Non-Cooperative Sensing
FDCS	Full-Duplex Cooperative Sensing
OFDM	Orthogonal Frequency Division Multiplexing
SINR	Signal-to-Interference-and-Noise-Ratio
CRN	Cognitive Radio Networks
CR	Cognitive Radio
PU	Primary User
SU	Secondary User
CG	Cooperative Gain
NP	Neyman–Pearson Criteron
